# Draft Genome Sequence of the Bacteriocin-Producing *Bradyrhizobium japonicum* Strain FN1

**DOI:** 10.1128/genomeA.00812-15

**Published:** 2015-07-30

**Authors:** MacLean G. Kohlmeier, Harry Yudistira, Xiang Li Zhang, Brian Fristensky, David B. Levin, Richard Sparling, Ivan J. Oresnik

**Affiliations:** a Department of Microbiology, University of Manitoba, Winnipeg, Manitoba, Canada; b Department of Plant Science, University of Manitoba, Winnipeg, Manitoba, Canada; c Biosystems Engineering, University of Manitoba, Winnipeg, Manitoba, Canada

## Abstract

*Bradyrhizobium japonicum* strain FN1 was found to produce bacteriocin-like zones of clearing when tested against other strains of bradyrhizbia. The genome was sequenced, and several putative bacteriocin-producing genes, in addition to the expected genes involved in nodulation and nitrogen fixation, were identified.

## GENOME ANNOUNCEMENT

*Bradyrhizobium japonicum* is a Gram-negative alphaproteobacterium that is capable of existing in either a free-living or a symbiotic state as a bacteroid in plant-derived organs, termed nodules, that develop on the root surface of the host organism *Glycine max*. Within nodules the bacteria reduce atmospheric nitrogen into an organic form that can be used by the plant for growth. For this reason, inocula of *B. japonicum* are commonly used in agriculture to increase crop yield. Rhizobial inoculums are often outcompeted for nodule occupancy by native strains present in the soil (1). Therefore, it is of interest to study factors with the potential to enhance the competitiveness of *B. japonicum* for nodule occupancy. Such factors include bacteriocins, which can be defined as narrow spectrum antibiotics produced by certain bacteria that are only effective against closely related strains (2). Bacteriocins have been shown to play a role in determining nodule occupancy in *Rhizobium leguminosarum* (3, 4).

Bacteriocins have been previously identified in *B. japonicum* (5), and we have isolated a bacteriocin-producing strain of *B. japonicum*, termed strain FN1, from Manitoba soils. In an effort to identify the genes responsible for bacteriocin production, the entire genome of FN1 was sequenced and annotated for further investigation.

The genome of *B. japonicum* strain FN1 was sequenced by the Next Generation Sequencing platform at the Manitoba Institute of Child Health using Illumina MiSeq technologies. Two successful runs both yielded 8,402,786 sequences, all of which were paired 150-bp reads with an average library insert size of 957 bp. Data output was assembled via Optimized-Velvet (6) into two formats; one consisting of 141 contigs and the other further organized into 87 scaffolds. Both data sets were submitted to the Joint Genome Institute’s (JGI) Integrated Microbial Genomes-Expert Review (IMG ER) platform (7) for annotation.

The genome consists of 9,138,496 bp, with a GC count of 64%, and has 8,613 coding sequences. It contains a symbiosis island, housing the nodulation and nitrogen fixation genes. Genes encoding the enzymes involved in the Calvin-Benson-Bassham cycle, as well as genes associated with hydrogen uptake, were also detected. This suggests that FN1 is capable of chemolithoautotrophic growth using H_2_ as an electron donor and CO_2_ as a source of carbon. In addition, characteristic genes of the Embden-Meyerhoff-Parnas pathway, the Entner-Doudoroff pathway, the pentose phosphate pathway, as well those involved in poly-hydoroxy alkanoate production, were present. These features are consistent with the published genomes of other *B. japonicum* strains (8–11).

To identify bacteriocin genes the genome was searched with BLAST (12) using the amino acid sequence of RTX-like toxins from *R. leguminosarum* as a query. Subsequent analysis was conducted with two Web-based software tools: antibiotics and Secondary Metabolite Analysis Shell (antiSMASH) (13) and BAGEL (14). These tools identified several putative bacteriocin-producing genes within the genome of FN1. Future work will involve mutating these genes to determine which of them confer the assayed bacteriocin activity.

### Nucleotide sequence accession numbers.

This whole-genome shotgun project has been deposited at DDBJ/EMBL/GenBank under the accession number JGCL00000000. The version described in this paper is the first version, JGCL01000000.
